# 3-Chloro-2-methyl­anilinium dihydrogenphosphate

**DOI:** 10.1107/S1600536808001700

**Published:** 2008-01-30

**Authors:** Hamed Khemiri, Samah Akriche, Mohamed Rzaigui

**Affiliations:** a Laboratoire de Chimie des Matériaux, Faculté des Sciences de Bizerte, 7021 Zarzouna Bizerte, Tunisia

## Abstract

The structure of the title compound, C_7_H_9_ClN^+^·H_2_PO_4_^−^, contains inorganic layers built by (H_2_PO_4_)^−^ anions and which are parallel to the *ab* planes around *z* = 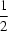. 3-Chloro-2-methyl­anilinium cations are anchored between the inorganic layers through N—H⋯O hydrogen bonds. Electrostatic and van der Waals inter­actions, as well as hydrogen bonds, maintain the structural cohesion.

## Related literature

For related literature, see: Adams (1977 ▶); Blessing (1986 ▶); Chtioui & Jouini (2004 ▶); Desiraju (1989 ▶, 1995 ▶); Hebert (1978 ▶); Oueslati & Ben Nasr (2006 ▶).
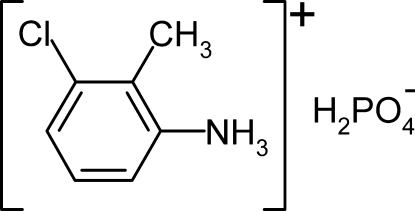


## Experimental

### 

#### Crystal data

C_7_H_9_ClN^+^·H_2_PO_4_^−^
*M* *_r_* = 239.59
Monoclinic, 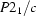

*a* = 16.942 (6) Å
*b* = 8.272 (2) Å
*c* = 7.979 (7) Å
β = 100.11 (5)°
*V* = 1100.8 (11) Å^3^
*Z* = 4
Mo *K*α radiation
μ = 0.48 mm^−1^
*T* = 292 K
0.40 × 0.30 × 0.20 mm

#### Data collection

Enraf–Nonius TurboCAD-4 diffractometer
Absorption correction: none
3304 measured reflections
1932 independent reflections
1736 reflections with *I* > 2σ(*I*)
*R*_int_ = 0.027
2 standard reflections frequency: 120 min intensity decay: 1%

#### Refinement

*R*[*F* ^2^ > 2σ(*F* ^2^)] = 0.044
*wR*(*F* ^2^) = 0.122
*S* = 1.08
1932 reflections
132 parameters
H-atom parameters constrained
Δρ_max_ = 0.36 e Å^−3^
Δρ_min_ = −0.38 e Å^−3^

### 

Data collection: *CAD-4 EXPRESS* (Enraf–Nonius, 1994 ▶); cell refinement: *CAD-4 EXPRESS*; data reduction: *XCAD4* (Harms & Wocadlo, 1995 ▶); program(s) used to solve structure: *SHELXS97* (Sheldrick, 2008 ▶); program(s) used to refine structure: *SHELXL97* (Sheldrick, 2008 ▶); molecular graphics: *ORTEP-3 for Windows* (Farrugia, 1997 ▶) and *DIAMOND* (Brandenburg & Putz, 2005 ▶); software used to prepare material for publication: *WinGX* (Farrugia, 1999 ▶).

## Supplementary Material

Crystal structure: contains datablocks I, global. DOI: 10.1107/S1600536808001700/dn2313sup1.cif

Structure factors: contains datablocks I. DOI: 10.1107/S1600536808001700/dn2313Isup2.hkl

Additional supplementary materials:  crystallographic information; 3D view; checkCIF report

## Figures and Tables

**Table 1:** Hydrogen-bond geometry (Å, °)

*D*—H⋯*A*	*D*—H	H⋯*A*	*D*⋯*A*	*D*—H⋯*A*
O1—H1⋯O3^i^	0.82	1.83	2.634 (3)	166
O2—H2⋯O3^ii^	0.82	1.83	2.596 (2)	154
N1—H1*A*⋯O3	0.89	2.04	2.886 (3)	158
N1—H1*B*⋯O4^iii^	0.89	1.84	2.722 (3)	172
N1—H1*C*⋯O4^ii^	0.89	1.84	2.708 (3)	166

Symmetry codes: (i) 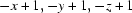; (ii) 
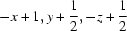
; (iii) 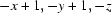.

**Fig. 1.**
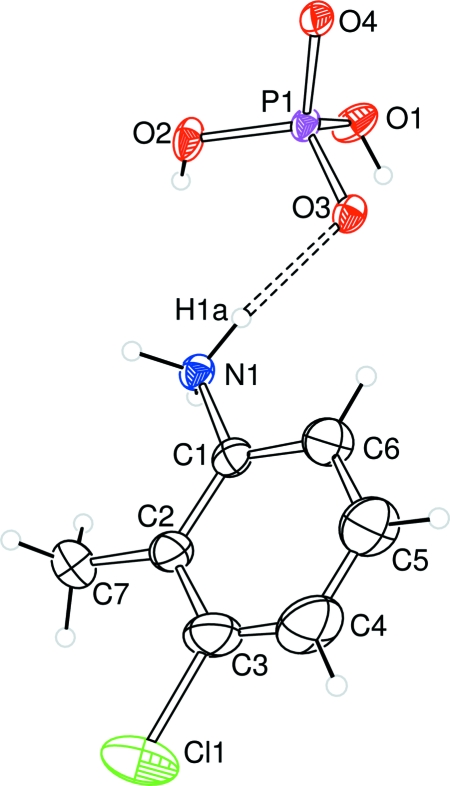
View of (I) with the atom-labelling scheme. Displacement ellipsoids are drawn at the 30% probability level. H atoms are represented as small spheres of arbitrary radii. H bond is shown as dashed line.

**Fig. 2.**
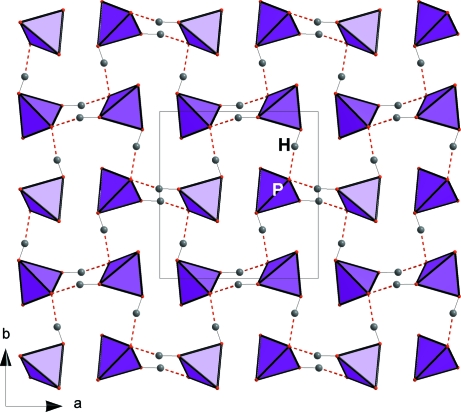
Projection of (H2PO4)n- subnetwork along c axis.

**Fig. 3.**
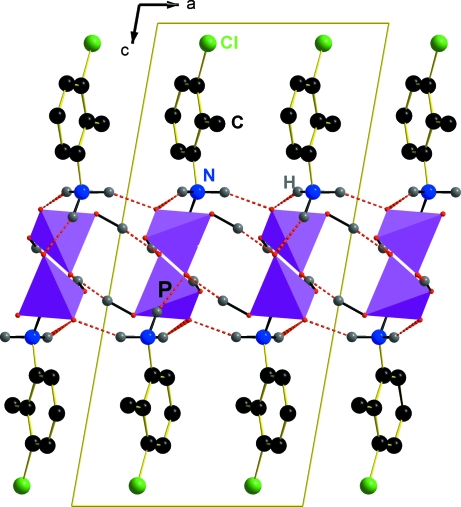
Projection of (I) along b axis.

## supplementary crystallographic information

### Comment

Inorganic-organic hybrid materials have been studied extensively because the blending of organic and inorganic components allows to the development of materials with novel properties. In particular the family of material which combine monophosphate anions with organic molecules such as aliphatic and aromatic amines has been studied intensively due to their numerous uses in various fields such as biomolecular science, liquid crystals, catalysts and fuel cells (Adams, 1977; Blessing, 1986; Desiraju, 1989, 1995). As a contribution to the study of this compound family, we report in this work the synthesis and the crystal structure of a new organic cation monophosphate [3-Cl-2-CH_3_C_6_H_3_NH_3_]H_2_PO_4_ (I).

The asymmetric unit in the structure of I consists of one phosphate anion (H_2_PO_4_)^-^ and one organic cation (3-Cl-2-CH_3_C_6_H_3_NH_3_)^+ ^(Fig. 1). A projection of the structure in the [001] direction shows that the (H_2_PO_4_) groups are interconnected by O—H···.O hydrogen bonds to form inorganic layers parallel to the (a, b) planes (Fig. 2). On both sides of each inorganic layer (Fig. 3), are grafted the organic cations compensating their negatives charges. Each (H_2_PO_4_) group is connected to three neighbours by strong hydrogen bonds, with O···O separations ranging from 2.596 (2) Å to 2.634 (3) Å (Table 1). Among the P—O distances in the PO_4_ tetrahedron, we can distinguish two different types: the shortest ones (1.498 (2) Å and 1.509 (2) Å) correspond to the phosphorus atom double bonded to oxygen atom (P=O); the largest ones (1.563 (2) Å and 1.564 (2) Å) can be attributed to P—OH distances. The average P—O distances and O—P—O bond angles are 1.534 Å and 109.42°, respectively, which fall in the range of the values observed in many phosphate materials (Hebert, 1978)). The strength of O—H···O hydrogen bond and the values of P···P distances (with a minimum value of 4.383 (4) Å) between two successive inorganic layers could allow us to consider the (H_2_PO_4_)_n_^-^ subnetwork as a polymeric species. Similar arrangement have been observed in other crystal structures (Chtioui & Jouini, 2004). The C—C bond lengths spreading between are 1.361 (6) and 1.500 (5) Å, which are between single and double bond and agree with that in 4-chloroanilinium dihydrogenmonophosphate (Oueslati & Ben Nasr, 2006).

### Experimental

Single crystals of the title compound (I) were prepared by adding drop by drop under stirring, an aqueous solution (10 ml) of orthophosphoric acid (0.25 mmol) (85 weight from Fluka %) to an alcoholic solution (10 ml) of 3-Cloro-2-methylaniline (0.15 mmol)(Across 98). The obtained solution was then slowly evaporated at room temperature until the formation of single crystals which were stable under normal condition of temperature and humidity.

### Refinement

All H atoms attached to C, N and O atoms were fixed geometrically and treated as riding with C—H = 0.93 Å (C_aromatic_) or 0.96 Å (C_methyl_), N—H= 0.89 Å and O—H = 0.82 Å and with *U*_iso_=1.2*U*_eq_(C_aromatic_,*O*) or *U*_iso_=1.5*U*_eq_(C_methyl_,*N*).

### Crystal data

**Table:**

C_7_H_9_ClN^+^·H_2_PO_4_^–^	*F*_000_ = 496
*M**_r_* = 239.59	*D*_x_ = 1.446 Mg m^−^^3^
Monoclinic, *P*2_1_/*c*	Mo *K*α radiation λ = 0.71073 Å
Hall symbol: -P 2ybc	Cell parameters from 25 reflections
*a* = 16.942 (6) Å	θ = 8–11º
*b* = 8.272 (2) Å	µ = 0.48 mm^−^^1^
*c* = 7.979 (7) Å	*T* = 292 K
β = 100.11 (5)º	Prism, colorless
*V* = 1100.8 (11) Å^3^	0.40 × 0.30 × 0.20 mm
*Z* = 4	

### Data collection

**Table:**

Enraf–Nonius TurboCAD4 diffractometer	*R*_int_ = 0.027
Radiation source: fine-focus sealed tube	θ_max_ = 25.0º
Monochromator: graphite	θ_min_ = 2.4º
*T* = 292 K	*h* = −10→20
non–profiled ω scans	*k* = −9→0
Absorption correction: none	*l* = −9→9
3304 measured reflections	2 standard reflections
1932 independent reflections	every 120 min
1736 reflections with *I* > 2σ(*I*)	intensity decay: 1%

### Refinement

**Table:**

Refinement on *F*^2^	Hydrogen site location: inferred from neighbouring sites
Least-squares matrix: full	H-atom parameters constrained
*R*[*F*^2^ > 2σ(*F*^2^)] = 0.044	*w* = 1/[σ^2^(*F*_o_^2^) + (0.0675*P*)^2^ + 0.6257*P*] where *P* = (*F*_o_^2^ + 2*F*_c_^2^)/3
*wR*(*F*^2^) = 0.122	(Δ/σ)_max_ = 0.011
*S* = 1.08	Δρ_max_ = 0.36 e Å^−^^3^
1932 reflections	Δρ_min_ = −0.38 e Å^−^^3^
132 parameters	Extinction correction: SHELXL97 (Sheldrick, 2008), Fc^*^=kFc[1+0.001xFc^2^λ^3^/sin(2θ)]^-1/4^
Primary atom site location: structure-invariant direct methods	Extinction coefficient: 0.015 (3)
Secondary atom site location: difference Fourier map	

### Special details

**Table:**

Geometry. All e.s.d.'s (except the e.s.d. in the dihedral angle between two l.s. planes) are estimated using the full covariance matrix. The cell e.s.d.'s are taken into account individually in the estimation of e.s.d.'s in distances, angles and torsion angles; correlations between e.s.d.'s in cell parameters are only used when they are defined by crystal symmetry. An approximate (isotropic) treatment of cell e.s.d.'s is used for estimating e.s.d.'s involving l.s. planes.
Refinement. Refinement of *F*^2^ against ALL reflections. The weighted *R*-factor *wR* and goodness of fit *S* are based on *F*^2^, conventional *R*-factors *R* are based on *F*, with *F* set to zero for negative *F*^2^. The threshold expression of *F*^2^ > σ(*F*^2^) is used only for calculating *R*-factors(gt) *etc*. and is not relevant to the choice of reflections for refinement. *R*-factors based on *F*^2^ are statistically about twice as large as those based on *F*, and *R*- factors based on ALL data will be even larger.

### Fractional atomic coordinates and isotropic or equivalent isotropic displacement parameters (Å^2^)

**Table:**

	*x*	*y*	*z*	*U*_iso_*/*U*_eq_	
P1	0.43820 (3)	0.46630 (6)	0.23711 (6)	0.0298 (2)	
O1	0.39492 (10)	0.5342 (2)	0.3800 (2)	0.0503 (5)	
H1	0.4278	0.5477	0.4674	0.075*	
O2	0.44438 (12)	0.60754 (19)	0.11002 (19)	0.0462 (5)	
H2	0.4562	0.6914	0.1634	0.069*	
O3	0.52069 (9)	0.40766 (18)	0.31601 (17)	0.0346 (4)	
O4	0.38537 (10)	0.34256 (19)	0.13619 (18)	0.0408 (4)	
Cl1	0.95781 (6)	0.6138 (3)	0.2672 (2)	0.1564 (8)	
N1	0.65051 (11)	0.5738 (2)	0.1985 (2)	0.0342 (4)	
H1A	0.6130	0.5026	0.2138	0.051*	
H1B	0.6435	0.6023	0.0893	0.051*	
H1C	0.6468	0.6609	0.2621	0.051*	
C1	0.72967 (14)	0.5010 (3)	0.2480 (3)	0.0392 (5)	
C2	0.79647 (15)	0.5918 (4)	0.2317 (3)	0.0524 (7)	
C3	0.86992 (18)	0.5110 (6)	0.2839 (5)	0.0784 (10)	
C4	0.8752 (2)	0.3579 (6)	0.3475 (6)	0.0954 (13)	
H4	0.9252	0.3102	0.3805	0.114*	
C5	0.8074 (2)	0.2742 (5)	0.3631 (6)	0.0875 (12)	
H5	0.8110	0.1701	0.4078	0.105*	
C6	0.73348 (18)	0.3460 (3)	0.3116 (4)	0.0617 (8)	
H6	0.6867	0.2900	0.3199	0.074*	
C7	0.7899 (2)	0.7623 (5)	0.1667 (5)	0.0807 (11)	
H7A	0.7432	0.8123	0.1966	0.121*	
H7B	0.7856	0.7619	0.0451	0.121*	
H7C	0.8368	0.8219	0.2168	0.121*	

### Atomic displacement parameters (Å^2^)

**Table:**

	*U*^11^	*U*^22^	*U*^33^	*U*^12^	*U*^13^	*U*^23^
P1	0.0413 (4)	0.0243 (3)	0.0221 (3)	−0.0007 (2)	0.0007 (2)	−0.00082 (18)
O1	0.0444 (9)	0.0731 (13)	0.0312 (9)	0.0113 (9)	0.0005 (7)	−0.0125 (8)
O2	0.0800 (12)	0.0248 (8)	0.0297 (8)	−0.0045 (8)	−0.0020 (8)	0.0030 (6)
O3	0.0448 (9)	0.0301 (8)	0.0276 (7)	0.0037 (6)	0.0023 (6)	−0.0020 (6)
O4	0.0548 (9)	0.0355 (9)	0.0285 (7)	−0.0119 (7)	−0.0026 (7)	0.0004 (6)
Cl1	0.0485 (6)	0.2330 (19)	0.1867 (15)	−0.0312 (8)	0.0178 (7)	0.0464 (14)
N1	0.0391 (10)	0.0352 (9)	0.0272 (8)	−0.0005 (8)	0.0029 (7)	−0.0024 (7)
C1	0.0416 (12)	0.0449 (12)	0.0305 (11)	0.0019 (10)	0.0048 (9)	−0.0055 (9)
C2	0.0451 (14)	0.0698 (18)	0.0419 (13)	−0.0073 (13)	0.0061 (11)	−0.0006 (13)
C3	0.0414 (15)	0.118 (3)	0.075 (2)	−0.0034 (18)	0.0094 (15)	0.001 (2)
C4	0.060 (2)	0.113 (3)	0.109 (3)	0.035 (2)	0.005 (2)	0.011 (3)
C5	0.072 (2)	0.066 (2)	0.120 (3)	0.0236 (18)	0.005 (2)	0.017 (2)
C6	0.0540 (15)	0.0458 (15)	0.083 (2)	0.0069 (13)	0.0066 (14)	0.0043 (14)
C7	0.075 (2)	0.085 (2)	0.079 (2)	−0.0277 (19)	0.0063 (18)	0.0238 (19)

### Geometric parameters (Å, °)

**Table:**

P1—O4	1.4980 (16)	C1—C2	1.383 (4)
P1—O3	1.5087 (17)	C2—C3	1.409 (4)
P1—O2	1.5626 (17)	C2—C7	1.500 (5)
P1—O1	1.5641 (19)	C3—C4	1.361 (6)
O1—H1	0.8200	C4—C5	1.365 (6)
O2—H2	0.8200	C4—H4	0.9300
Cl1—C3	1.740 (4)	C5—C6	1.382 (4)
N1—C1	1.460 (3)	C5—H5	0.9300
N1—H1A	0.8900	C6—H6	0.9300
N1—H1B	0.8900	C7—H7A	0.9600
N1—H1C	0.8900	C7—H7B	0.9600
C1—C6	1.377 (4)	C7—H7C	0.9600
			
O4—P1—O3	115.27 (9)	C3—C2—C7	123.8 (3)
O4—P1—O2	105.31 (10)	C4—C3—C2	123.3 (3)
O3—P1—O2	110.39 (10)	C4—C3—Cl1	118.8 (3)
O4—P1—O1	108.96 (11)	C2—C3—Cl1	117.9 (3)
O3—P1—O1	109.25 (10)	C3—C4—C5	120.3 (3)
O2—P1—O1	107.34 (11)	C3—C4—H4	119.9
P1—O1—H1	109.5	C5—C4—H4	119.9
P1—O2—H2	109.5	C4—C5—C6	119.2 (4)
C1—N1—H1A	109.5	C4—C5—H5	120.4
C1—N1—H1B	109.5	C6—C5—H5	120.4
H1A—N1—H1B	109.5	C1—C6—C5	119.4 (3)
C1—N1—H1C	109.5	C1—C6—H6	120.3
H1A—N1—H1C	109.5	C5—C6—H6	120.3
H1B—N1—H1C	109.5	C2—C7—H7A	109.5
C6—C1—C2	123.7 (3)	C2—C7—H7B	109.5
C6—C1—N1	117.7 (2)	H7A—C7—H7B	109.5
C2—C1—N1	118.6 (2)	C2—C7—H7C	109.5
C1—C2—C3	114.2 (3)	H7A—C7—H7C	109.5
C1—C2—C7	122.1 (3)	H7B—C7—H7C	109.5

### Hydrogen-bond geometry (Å, °)

**Table:**

*D*—H···*A*	*D*—H	H···*A*	*D*···*A*	*D*—H···*A*
O1—H1···O3^i^	0.82	1.83	2.634 (3)	166
O2—H2···O3^ii^	0.82	1.83	2.596 (2)	154
N1—H1A···O3	0.89	2.04	2.886 (3)	158
N1—H1B···O4^iii^	0.89	1.84	2.722 (3)	172
N1—H1C···O4^ii^	0.89	1.84	2.708 (3)	166

Symmetry codes: (i) −*x*+1, −*y*+1, −*z*+1; (ii) −*x*+1, *y*+1/2, −*z*+1/2; (iii) −*x*+1, −*y*+1, −*z*.
